# Multi-b-value diffusion stretched-exponential model parameters correlate with MIB-1 and CD34 expression in Glioma patients, an intraoperative MR-navigated, biopsy-based histopathologic study

**DOI:** 10.3389/fonc.2023.1104610

**Published:** 2023-04-26

**Authors:** Junlong Wang, Hua Zhang, Xuefei Dang, Wenting Rui, Haixia Cheng, Jing Wang, Yong Zhang, Tianming Qiu, Zhenwei Yao, Hanqiu Liu, Haopeng Pang, Yan Ren

**Affiliations:** ^1^ Department of Radiology, Huashan Hospital, Fudan University, Shanghai, China; ^2^ Department of Radiology, the Affiliated Hospital of Qingdao University, Qingdao University, Qingdao, China; ^3^ Department of Oncology, Minhang Branch of Fudan University Shanghai Cancer Center, Fudan University, Shanghai, China; ^4^ Department of Neuropathology, Huashan Hospital, Fudan University, Shanghai, China; ^5^ Department of Magnetic Resonance Research, General Electric Healthcare, Shanghai, China; ^6^ Department of Neurosurgery, Huashan Hospital, Fudan University, Shanghai, China; ^7^ Minimally Invasive Therapy Center, Shanghai Cancer Center, Fudan University, Shanghai, China; ^8^ Department of Radiology, Ruijin Hospital, Shanghai Jiao Tong University, Shanghai, China

**Keywords:** glioma, diffusion magnetic resonance imaging, stretched exponential model, biopsy, intraoperative navigation

## Abstract

**Background:**

To understand the pathological correlations of multi-*b*-value diffusion-weighted imaging (MDWI) stretched-exponential model (SEM) parameters of α and diffusion distribution index (DDC) in patients with glioma. SEM parameters, as promising biomarkers, played an important role in histologically grading gliomas.

**Methods:**

Biopsy specimens were grouped as high-grade glioma (HGG) or low-grade glioma (LGG). MDWI-SEM parametric mapping of DDC_1500_, α_1500_ fitted by 15 *b*-values (0-1,500 sec/mm^2^)and DDC_5000_ and α_5000_ fitted by 22 *b*-values (0-5,000 sec/mm^2^) were matched with pathological samples (stained by MIB-1 and CD34) by coregistered localized biopsies, and all SEM parameters were correlated with these pathological indices pMIB-1(percentage of MIB-1 expression positive rate) and CD34-MVD (CD34 expression positive microvascular density for each specimen). The two-tailed Spearman’s correlation was calculated for pathological indexes and SEM parameters, as well as WHO grades and SEM parameters.

**Results:**

MDWI-derived α_1500_ negatively correlated with CD34-MVD in both LGG (6 specimens) and HGG (26 specimens) (r=-0.437, *P* =0.012). MDWI-derived DDC_1500_ and DDC_5000_ negatively correlated with MIB-1 expression in all glioma patients (*P*<0.05). WHO grades negatively correlated with α_1500_(r=-0.485; *P=*0.005) and α_5000_(r=-0.395; *P=*0.025).

**Conclusions:**

SEM-derived DDC and α are significant in histologically grading gliomas, DDC may indicate the proliferative ability, and CD34 stained microvascular perfusion may be an important determinant of water diffusion inhomogeneity α in glioma.

## 1 Introduction

Gliomas are the most common primary malignant central nervous system cancer, accounting for 80%-85% of malignant brain tumors (1), being highly aggressive and having the highest mortality and morbidity. The current standard of care treatment for glioma is maximal safe surgical resection followed by radiation therapy and concomitant chemotherapy (2). Despite the mainstream multimodal treatment, the median survival of most malignant glioblastoma patients has only improved to approximately 15 months (3), and less than 10% survive for over five years (4). According to the 2021 World Health Organization (WHO) criteria, gliomas are classified as grades I-IV (5), with different molecular subtypes and histopathology; these intrinsic subtypes showed different prognoses and outcomes (5). There exists a remarkable disparity in clinical treatment and prognosis between the different grades of glioma (3, 6). Thus, the preoperative differential diagnosis is essential for therapeutic decisions and determining the prognosis of patients with glioma.

MRI is the main workhorse for the evaluation of intracranial glioma (7). Modern diffusion MR protocols can acquire the diffusion data in a reasonable operating time and visualize the brain tissue *in vivo* at the micrometer scale. In a recent survey from Europe, beyond dynamic contrast enhanced (DSC)-MRI (67.6%), diffusion MRI (82.0%) was the most frequently used quantitative MR imaging technique in clinical neuroradiological practice (7). As yet, the apparent diffusion coefficient (ADC) from mono-exponential DWI, as an imaging biomarker, is the most frequently used diffusion parameter for tumor differentiation and monitoring of treatment efficacy (8). However, the mono-exponential model is susceptible to the microcirculation of blood in capillaries with inherent limitations in measuring the non-Gaussian distribution of water molecules. Le Bihan et al. (9) proposed the biexponential intravoxel incoherent motion (IVIM) DWI model with its aim to allow separation of molecular water diffusion from microcirculation, and to facilitate glioma differential diagnosis as well as identify the subtype of IDH mutation in gliomas (10). Moreover, there is evidence that the water molecular diffusion heterogeneity index (α) of the stretched exponential model (SEM) exhibits higher differential diagnosis sensitivity (92.9%) and specificity (100%) in grading glioma (11). Similarly, Zhang et al. also found that α showed the highest grading efficacy of glioma in their studies (12). Besides, there is evidence that diffusion distribution index (DDC), the other parameter from the SEM, is valuable in differentiating high-grade glioma (HGG) from low-grade glioma (LGG) compared to ADC (13). Chen et al. also suggested that DDC showed the highest sensitivity and specificity for glioma grading (14). Although the clinical values of various diffusion MRI models have been well proved in glioma differentiation by many researchers, few studies have explained the underlying histopathological significance under these parameters.

Tumor histopathology is the golden standard for the evaluation of glioma malignancy. According to the WHO guidelines, the following histological criteria were used to grade gliomas: cytological atypia, mitotic activity, cellularity, microvascular proliferation and/or necrosis (5). Mitotic activity is helpful in the distinction of grade II and grade III gliomas, whereas microvascular proliferation may suggest high-grade gliomas of grade III or grade IV (5). Some well-known ancillary markers of grading gliomas, such as anti-MIB-1/Ki-67 and anti-CD34, are closely related to tumor cell proliferation and microvascular density (5, 15). Anti-CD34 is a marker of vascular endothelial cells with high sensitivity and specificity of small and large vessels in both normal and tumor tissue (15). Furthermore, quite a few correlation studies have proved that DWI-related parameters are the potential non-invasive surrogates of histopathologic indexes, such as tumor cell density (16) and proliferative activity (17). Over the past decade of clinical research, IVIM-DWI derived parameters may simultaneously reflect tumor histology of microvessel density (MVD) or the percentage of MIB-1 expression positive rate(pMIB-1) both in humans and animals (18). Meanwhile, VERDICT MR with multiple b-value diffusion technique has been developed to reflect cell size (19, 20). However, although SEM-DWI derived parameters with the best differential performance (with highest AUC value of 0.968) in the differentiation of grades III from IV (14) for DDC and with the highest AUC of 0.993 in the differentiation of HGG and LGG (11) for heterogeneity index α, under which the exact correlation with histopathological biomarkers has rarely been reported (14).

However, due to not an exact match of tumor specimen and delineated tumor region of interest (ROI) on DWI images, prior studies’ correlation results have inherent limitations in identifying the DWI parameters as imaging biomarkers, which do not necessarily correspond to regions manifested on MRI images. Thus, the method of using the spatial location of each biopsy target based on a scanning coordinate system in an MR unit should be employed to improve the accuracy of the correlation study (21). This study correlated histopathological indexes with SEM-DWI parameters based on an intraoperative MR-navigated neurosurgical biopsy by a scanning coordinate system matching histopathological indexes and imaging parameters in gliomas.

## 2 Article types

Retrospective diagnostic study performed at one institution

## 3 Materials and methods

This retrospective study was approved by the institutional Ethics Committee of our hospital.

### 3.1 Participants

Subjects were eligible for our study if they were diagnosed with cerebral gliomas by histopathological biopsy and ineligible if they had any other types of tumors or any treatments before. Twenty-one glioma patients (fourteen males and seven females, 10–65 years old, 30-80kg) were respectively enrolled from July 2014 to January 2017. All patients underwent multi-b-value DWI examination with 22 b values from 0-5,000 sec/mm^2^), followed by MRI-guided stereotactic biopsy and/or surgical resection. Participants were classified two groups (LGG and HGG) according to the WHO classification, randomization group method was not used in this study.

### 3.2 Image data acquisition

All patients underwent imaging using a 3T MR imaging unit (Discovery MR 750; GE Medical Systems, Milwaukee, Wis) and an eight-channel head coil (GE Medical Systems). The protocol of MR examination included the following sequences: axial T1WI (TR/TE:3195ms/24ms; Matrix: 256x256; section thickness (ST)/gap: 4mm/0 mm), T2WI (TR/TE:9185ms/108ms; matrix: 256x256; ST/gap: 4mm/0 mm), T2 fluid-attenuated inversion recovery (T2-FLAIR) (TR/TE: 9491ms/140ms, Matrix: 256×256; ST/gap: 4mm or 2mm/0 mm), and 3D T1WI with gadolinium-enhanced (3D-T1C) (TR/TE: 8.2ms/3.2ms, Matrix: 256×256; ST/gap: 1mm/0 mm). MDWI sequence was performed by using a single-shot echo-planar sequence in the axial plane, (TR/TE:4000ms/90.6ms, ST/gap:4mm/0 mm, field of view of 24cm, and matrix of 128×128 with total 22 *b* values from 0 to 5000 sec/mm^2^ (0, 10, 20, 30, 50, 100, 150, 200, 300, 400, 500, 600, 800, 1,000, 1,500, 2,000, 2,500, 3,000, 3,500, 4,000, 4,500, and 5,000 s/mm^2^, NEX increases with the *b* value, set from 1 to 4) in three diffusion directions. Total acquisition time is 7 mins. Scanning parameters are presented in **Table 1**.

**Table 1:** Main MRI sequences and parameters.

Sequence	TR/TE (ms)	FA	Matrix	FOV (mm^2^)	ST (mm)	Acquisition time (min:s)
eDWI	4000/90.6	90	128×128	240	4	7:00
T2-FLAIR	9491/140	111	256×256	240/256	4/2*	2:33; 6:50
3D-T1C	8.2/3.2	12	256×256	240/256	4/1*	1:40; 3:45-4:20

TR/TE, repetition time/echo time; FA, flip angle; FOV, field of view; ST, Slice thickness; * indicates a slice thickness of 4 mm for routine scan, and 2 mm for T2-FLAIR or 1 mm for 3D-T1C used for the iMR navigation sequence.

### 3.3 iMRI-navigated biopsy

**Figure 1:**
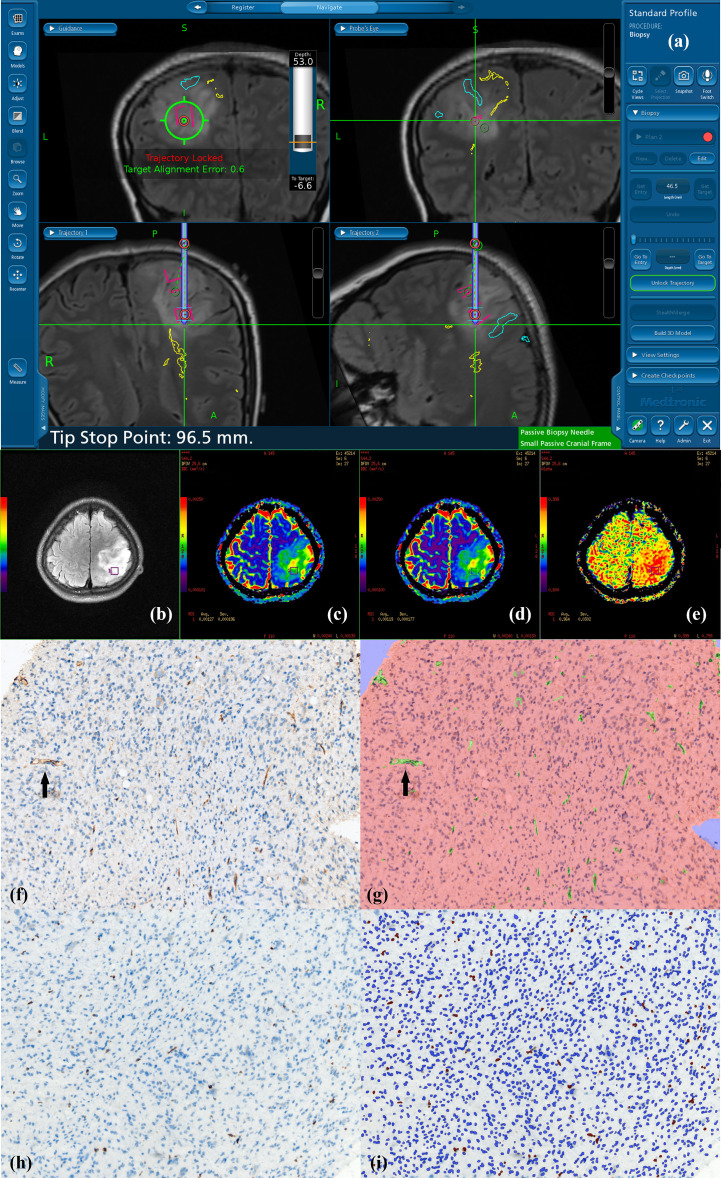
Navigated biopsy in a 31-year-old woman with pathologically confirmed WHO grade II astrocytoma. **(A)** Preoperative T2-FLAIR imaging was used for MR-guided biopsy; one specimen was obtained within the marked target regions (red circle) from the solid partition of tumor. **(B)** Target ROI taking the biopsy target coordinates as the center is shown on axial T2-FLAIR imaging (purple square in B). **(C)** DDC_1500_ mapping with the biopsy target (ROI: green square) **(D)** DDC_5000_ mapping with the biopsy target (ROI: green square) **(E)** α_1500_ mapping with the biopsy targets (ROI: green square). **(F)** AntiCD34 immunohistochemical staining (original magnification ×200 for all stains). **(G)** Computer assisted quantification is used to outline and calculate the neovascularization area in green. Black arrow shows microvasculature stained with CD34. **(H)** MIB-1 expression by using a high-power objective with a magnification of 200×. Immunohistochemical dying of specimen from the biopsy target showed MIB-1 expression, proliferation indexes were 4.02%. **(I)** Computer assisted quantification is used to outline and calculate the MIB-1 expression area in brown.

Each patient underwent a stereotactic puncture biopsy using a navigation system (Medtronic StealthStation i7 Integrated Navigation System; Canada). The region of interest (ROI) (“biopsy target”) was chosen by consensus of a neurosurgeon and a neuroradiologist with 15 and 13 years of experience, respectively. ROI for puncture was selected on 3D-T1C when the glioma showed noticeable enhancement or T2-FLAIR was selected when the tumor showed little or no enhancement. Each ROI was located in a solid part of the tumor, with dimensions of 10 × 10 × 10 mm. The region was marked with a highlighted frame by SINORAO medical workstation (SINORAO; Shenzhen, China) on anatomical 3D-T1C or T2FLAIR images. The centric coordinate of each ROI was recorded. The anatomical 3D-T1C or T2-FLAIR images with marked ROI for biopsy were transferred to the navigation system. A biopsy needle with a side-cut of 9.0×2.2 mm was used to obtain samples. Intraoperative MRI or postoperative computed tomography (CT) was used to ensure the accuracy of the sampling (**Figure 1**). The iMR-navigated biopsy process was also described in our prior study (21).

### 3.4 Imaging data analysis and processing

All images were obtained and transferred to a workstation (Advantage Workstation 4.6; GE Medical Systems) for processing. They were independently processed by two neuroradiologists (Y.R and H.Z) who were blinded to the histopathologic results for processing. By using a stretched exponential model for multi-*b*-value DWI, the water molecular diffusion heterogeneity index α and the distributed diffusion coefficient (DDC) were obtained by using the following method:


$$\frac{Sb}{S\left(0\right)}=\;\text{exp}\left[-(b\cdot DDC{)}^{\alpha }\right]$$

In this equation, α represents the intravoxel water molecular diffusion heterogeneity, which varies from 0 to 1 which can be considered a measure of the average difference between apparent water diffusion rates in a single voxel. A higher α value indicates lower intravoxel diffusion heterogeneity (approaching the mono-exponential decay), and the DDC represents the mean intravoxel diffusion rate (22). Two methods of selecting b-values were applied for fitting the stretched exponential model with 15 (0, 10, 20, 30, 50, 100, 150, 200, 300, 400, 500, 600, 800, 1,000, 1,500 sec/mm^2^) of 22 *b* values and all 22 b values, respectively. After the fitting procedure, four parametric maps were generated, two for a b-value range of 0–1,500 s/mm^2^ (DDC_1500_ and α_1500_) and two for a *b*-value range of 0–5,000 s/mm^2^ (DDC_5000_ and α_5000_).

Take axial three-dimensional T1 contrast (3D-T1C) or T2 fluid attenuated inversion recovery (FLAIR) images as reference. According to the recorded centric coordinate of each ROI, tumor ROIs with an area of 90–120 mm^2^ were placed on axial 3D-T1C or T2FLAIR images in each patient, and the measurements of α and DDC were automatically calculated. All image processing and analysis were performed using the in-built MADC module on the workstation (Advantage Workstation 4.6; GE Medical Systems). Parametric measurements (DDC_1500_, α_1500_, DDC_5000_ and α_5000_) were performed by two experienced radiologists (HZ and YR), respectively, who were blinded to the pathologic diagnosis. The mean values of these measurements were used for the correlation analysis.

### 3.5 Pathological data analysis and processing

Surgical biopsy specimens were embedded in paraffin and sliced into 4-μm sections. Hematoxylin and eosin (H&E)-stained sections were graded based on the 2016 edition World Health Organization (WHO) classification of central nervous system (CNS) tumors by a neuropathologist with 25 years of experience who was blinded to imaging results. Immunohistochemical sections were stained by anti-CD34 and anti-Ki67 (Changdao Bio-Reagent Company, Shanghai, China) monoclonal antibodies to calculate CD34-MVD and MIB1-LI, respectively. A multispectral phenotyping microscope (PerkinElmer, Vectra, Boston, MA) was used to photograph and quantitatively analyze CD-34 and MIB-1 expression. Image analysis software (Inform v. 2.01, PerkinElmer) was used to recognize the wavelengths for positive and negative cells in the section according to the standard spectrum.

CD34 antigen-stained microvessels in all tumor fields (total 3-20 fields for each slice) were counted, and the tissue area for each field was measured. The tumor microvasculature was assessed at ×200 magnification. CD34-MVD was defined according to the following formula: C = A/B, where A is the number of CD34-labelled microvessels in all specimen fields, and B is the total area of the same specimen fields, and C is the mean CD34-MVD per field.

The percentage of MIB-1-positive cells per field was calculated as MIB-1-positive expression levels according to the following formula: C = A/B ×100%, where A is the sum of MIB-1-positive cells in all specimen fields, B is the number of specimen fields, and C is the mean MIB-1-positive cells per field. All fields (total 3-20 fields for each slice) in each specimen were detected consecutively using high magnification (×200).

### 3.6 Statistical analysis

All statistical analyses were performed with SPSS (Version 27.0, IBM, Armonk, NY, USA; RRID: SCR_002865), and *P* values< 0.05 were considered statistically significant. The intraclass correlation coefficient (ICC) was calculated to assess the interobserver variation. ICC ranges between 0.0 and 1.0, and the inter-observer agreement was defined as follows:<0.40, poor; 0.40–0.60, moderate; 0.61– 0.80, good; >0.80, excellent. Spearman’s correlation analysis was used to evaluate the correlation of the between the variables: ISEM-derived parameters (DDC_1500_, α_1500_, DDC_5000_ and α_5000_) and histopathological indexes of CD34-MVD and pMIB-1. ②SEM-derived parameters (DDC_1500_, α_1500_, DDC_5000_ and α_5000_) and glioma WHO grades. Correlation coefficient Rho (r) was classified as little or fair (r ≤ 0.40), moderate to good (0.40< r ≤ 0.75), and very good to excellent (r > 0.75) (12).

## 4 Results

### 4.1 Patient groups and tissue samples

Total 32 specimens of gliomas from 21 patients (male: 14, female: 7, and median age of 51 ranging from 10 to 65 years old) were enrolled, which were confirmed by post-surgical histopathology, were classified as WHO Grade II (n=6), Grade III(n=18) and Grade IV(n=8). Six specimens of gliomas from 6 patients with WHO grade II located in the frontal lobe (n=4), insular lobe(n=1) and cerebellum(n=1), 4 of 6 patients underwent only surgical biopsy and the other two received biopsy followed by total tumor resection. Eighteen specimens of 11 patients (each patient with 1 specimen for 6 patients, each patient with 2 specimens for 4 patients and one patient with 4 specimens) with WHO grade III located in the frontal lobe (n=4), frontal parietal lobe (n=1), temporal lobe(n=2), cerebellum(n=1), basal ganglia(n=2) and thalamus(n=8), 13 of which underwent only biopsy, three received biopsies followed by subtotal resection, and the other two received biopsy followed by total resection. Eight specimens of gliomas from 4 patients (two specimens for each patient) with WHO grade IV located in the frontal lobe (n=2), parietal lobe (n=2), temporal lobe (n=2), and corpus callosum (n=2), 3 of 4 patients underwent only biopsy and the other one received biopsy followed by total resection.

### 4.2 Correlation of DDC and α with glioma grades

**Figure 2:**
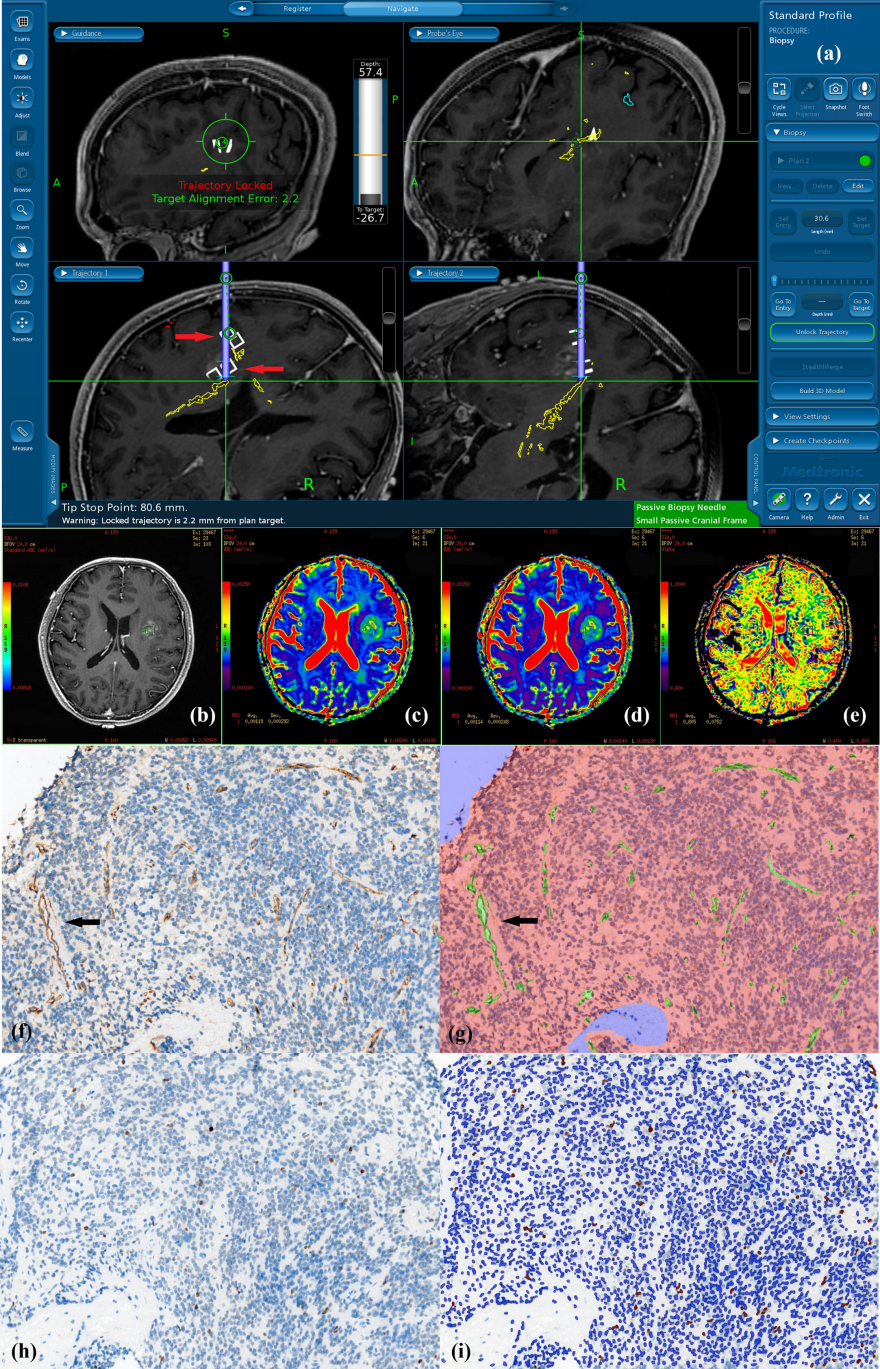
Navigated biopsy in a 57-year-old man with pathologically confirmed WHO grade III astrocytoma. **(A)** Preoperative enhanced T1WI imaging was used for MR-guided biopsy; two specimens were obtained within the marked target regions (two white square pointed by red arrow on the biopsy pathway) from the solid partition of tumor. **(B)** One deeper target ROI taking the biopsy target coordinate as the center is shown on axial T1C imaging within the contrast-enhance tumor (green square in B). **(C)** DDC_1500_ mapping with the biopsy target (ROI: green square) **(D)** DDC_5000_ mapping with the biopsy target (ROI: green square) **(E)** α_1500_ mapping with the biopsy target (ROI: green square). **(F)** AntiCD34 immunohistochemical staining (original magnification ×200 for all stains). **(G)** Computer assisted quantification is used to outline and calculate the neovascularization area in green. Arrows show microvasculature stained with CD34. **(H)**MIB-1 expression by using a high-power objective with a magnification of 200×. Immunohistochemical dying of specimen from the biopsy target showed MIB-1 expression, proliferation indexes were 2.22%. **(I)** Computer assisted quantification is used to outline and calculate the MIB-1 expression area in brown.

WHO grades negatively correlated with both α_1500_ (r =-0.485; *P=*0.005) and α_5000_ (r =-0.395; *P=*0.025) (**Figure 2**). No significant correlation found between DDC_1500_ and WHO grades, as well as DDC_5000_ and WHO grades (**Table 2**).

**Table 2:** Correlations of SEM-derived parameters with MIB-1, CD34, and WHO grades.

		DDC_1500_	DDC_5000_	α_1500_	α_5000_
pMIB-1	Correlation Coefficient	-0.397^*^	-0.410^*^	-0.165	-0.165
	Sig. (2-tailed)	0.027	0.025	0.374	0.374
	N	31	31	31	31
CD34-MVD	Correlation Coefficient	-0.296	-0.312	-0.437^*^	-0.437^*^
	Sig. (2-tailed)	0.101	0.082	0.012	0.012
	N	32	32	32	32
WHO grades	Correlation Coefficient	-0.280	-0.272	-0.485^**^	-0.395^*^
	Sig. (2-tailed)	0.120	0.133	0.005	0.025
	N	32	32	32	32

**. Correlation is significant at the 0.01 level (2-tailed).

*. Correlation is significant at the 0.05 level (2-tailed).

### 4.3 Correlation of DDC and α with pMIB-1 and CD34-MVD

The pMIB-1 for all glioma samples negatively correlated with DDC_1500_ (r =-0.397; *P=*0.0269, and DDC_5000_(r = -0.410, *P* =0.0254). There was a moderate to good negative correlation for all gliomas between CD34-MVD with α_1500_ (r =-0.437; *P* =0.012). Otherwise, the correlations between these parameters and pathological indexes and shown in table. And no significant correlation found between α_5000_ and histopathological indexes of CD34-MVD and pMIB-1 (**Table 2**).

## 5 Discussion

In this study, SEM-derived intravoxel water diffusion heterogeneity α had a moderately to good negative correlation with CD34 expression rate in glioma by MDWI using a narrow-low range of multi-*b*-value combination. Meanwhile, we also observed a suggestive negative correlation between α and WHO grades. As such, α may serve as a potential indicator in identifying the malignant part of the intratumor by reversely indicating the CD34-stained vascularity. Meanwhile, SEM-derived DDC showed a significant negative correlation with pMIB-1, which may help non-invasively assess gliomas’ tumor proliferation.

As far as we know, many diffusion models, such as DKI, DTI, IVIM and FROC, have been analyzed for the correlations between diffusion parameters and pathological biomarkers in the prior studies on human gliomas. For example, DKI derived parameters correlated with the diverse nuclear-to-cytoplasmic ratio, which can be used to distinguish primary central nervous system lymphoma and high-grade glioma (23); and DTI parameters were ever discussed about their correlations with proliferation and microvascular density (24). IVIM biexponential parameters were recently discussed about the correlations between IVIM-derived fast diffusion and perfusion fraction parameters and VEGF- and MIB-1-positive rates in brain gliomas (25). However, despite of the usefulness of SEM parameters in grading gliomas (26, 27), they have not been analyzed on the correlations between the parameters and histological biomarkers in gliomas. Theoretically, the SEM derived heterogeneity index α is promising to indicate the intratumor’s water diffusion heterogeneity of gliomas according to its definition as an indicator of water diffusion heterogeneity index.

Several studies have suggested the particular usefulness of this SEM-derived intravoxel heterogeneity index α in grading gliomas (26, 27). Higher intravoxel diffusion heterogeneity with a lower α value in HGG than LGG is a well-known fact reported in prior studies (11, 28). Theoretically, the function of the stretched exponential model, also known as the Kohlrausch-Williams-Watts model (29), has been widely used in other fields of physics. From 2003 to 2004, this model was successfully introduced by Bennett and applied to the study of animal brain tissue and glioma model for nearly 20 years (30, 31). SEM-derived α enables the invasively quantitative evaluation of intravoxel heterogeneity in the distribution of diffusion coefficients for brain tissues and gliomas (30), which may not only identify normal brain cortex from white mater fiber in rat brain but distinguish the invading tumor cells in the peritumoral edema area surrounding the contrast-enhanced gliomas (30, 31). However, the exact biophysical implications of SEM-derived α remain to be elucidated in human brain gliomas, despite theoretically tortuous vasculature and heterogeneous cellular morphology presumed to be the leading causes of intravoxel heterogeneity to Bennett’s assumption (30). Through this practice-focused research, we confirmed our hypothesis that the performance of α have great association with clinical pathology, but still further studies are needed to confirm our results.

In this study, the heterogeneity index α was observed with a significant inverse correlation with anti-CD34 labelled vascularity in all gliomas. One possible explanation of such inverse correlation is that decreased α values indicates higher intravoxel diffusion heterogeneity, which is consistent with the characteristic feature of glioblastoma Anti-CD34 is a marker of vascular endothelial cells of small and large vessels in normal and tumor tissue (15, 32). Prior studies by Kong et al. (33) and Rahmah et al. (34) also suggested that CD34 overexpression is associated with higher grades of gliomas. It is well known that tumor areas with active vascular proliferation are often synchronized with tumor proliferation, and the primary tumor mass cannot grow beyond the size of about 1 - 2 mm without recruiting local host vasculature (22) or the formation of new vessels by angiogenesis (34). Meanwhile, to ensure the accuracy of pathological diagnosis, preoperative puncture targets determined by MR enhancement or T2FLAIR are often considered the most active tumor growth sites with an abundant tumor cell and vascular proliferation activity (21). Therefore, the abundance of CD34 - labelled microvessels represents the most malignant tumor area, which may be reflected using the heterogeneity index α. Therefore, as a reflection of the intravoxel heterogeneity in the distribution of diffusion coefficients for malignant glioma, α may not only serve as a useful imaging biomarker in helping glioma grading, but also a valuable imaging index of glioma heterogeneous assessment. Besides, there is evidence that tumor heterogeneity is a possible explanation of some observed drug-resistance (35), so α may also contribute to drug-resistance prediction.

Significant negative correlations were observed between DDC and pMIB-1 in grade II-IV gliomas regardless of the multi-*b*-value range (15 *b* values for 0-1500 s/m^2^) and 22 *b* values *for* 0-5000 s/m^2^) in this study. Previous researchers have ever performed a direct comparison of ADC values with the MIB-1(Ki-67), which demonstrated variable results from significant or moderately inverse correlation (28, 35) between ADC values and MIB-1 in brain gliomas. However, the mono-exponential model-derived ADC value, calculated by the assumption of voxels composed of one proton pool, was nonideal due to intratumor inherent heterogeneity when the number of contributing proton pools is unknown. Theoretically, DDC can be considered an ADC approximation weighted by the volume fraction of water in each part of the continuous distribution, and previous study conducted by Chen et al. found that DDC manifests stronger correlation with Ki-67 LI than the ADC derived from monoexponential model DWI (14). Previous studies (14, 36) have also performed the correlation analysis between DDC and pMIB-1. Moreover, to the best of our knowledge, most studies have ever performed the comparison between DDC and pMIB-1 by using the resected or biopsied samples, none of which strictly bridged their connection by point-to-point spatial coordinates in glioma patients. Due to the inherent intratumor heterogeneity in glioma (37), the resected tumor samples can’t be accurately matched with presurgical MR imaging parameters. The corresponding results varied widely and were hardly convincing in guiding the clinical practice. In this study, the center coordinates of the biopsy target were confirmed according to presurgical 3D-T1C (1mm in ST) or T2FLAIR (2mm in ST) images with prelabeled markers on the scalp for intraoperative MR navigation. Then, by the inbuilt software in the workstation, the coordinates of the selected biopsy were put into and labelled into the 3D-T1C or T2FLAIR navigation sequence to guide the following surgical biopsy. At last, on the SEM-derived DDC and α map fused with 3D-T1C or T2FLAIR, a square ROI with 90-120(mm^2^) in size, centered by the selected coordinates for biopsy, was placed on the tumor biopsy area of 3D-T1C or T2FLAIR. Compared to prior studies (38, 39), the matching of biopsied specimens and imaging ROI of the tumor was considered to a maximal extent. Meanwhile, the pMIB-1 was calculated using a multispectral phenotyping microscope and imaging analysis software, which ensured the accuracy of measurement to a maximum extent. Thus, the precise correlation analysis between DDC and pMIB-1 in glioma patients was of great significance in improving the understanding of the biophysical implications of DDC.

In this study, we chose DDC to correlate with pMIB-1 and reached consistent results with previous studies which used ADC as their variables. One possible explanation that could be attributed to such a phenomenon is that increased pMIB-1 indicates active cell replication and division, which indirectly reflects higher cellularity and malignancy, leading to the decrease of intercellular gaps and increase of diffuse coefficient. As a reflection of the distribution of diffusion coefficient, DDC calculated from the stretched-exponential model may also be affected.

Several limitations of this study should be considered in interpreting our findings. First, a multi-b-value DWI sequence based on 22 *b* values takes a longer scan time of 7 mins, which may lead to the failure of data acquisition due to the patients’ motion in clinical practice and requires to be improved in future work by reducing the number of b-values from 22 to 15 and lowering repetition time from 4000ms to 3000ms. Especially for those seriously ill patients, it was difficult for them to stay calm during the whole scanning process to obtain reliable data. Second, at the skull base, the measurements of DWI-MRI parameters on the tumor area may be inaccurate due to image distortion from inherent deficits of diffusion sequence. Although all the badly deformed cases and those with severe motion disturbance have been excluded in the present study, a slight distortion could not be completely avoided. Third, relatively small samples could bias the results, especially for the low-grade glioma samples, due to less frequency of clinical demand for surgical biopsy. Fourth, white matter tracts in the tumor contour were not reconstructed and analyzed, as the fiber tracts can significantly influence the diffusion parameters when the fibrous myelin sheath in the tumor remains intact, which should be considered in the future validation study. Last, although accurate matching method employed in the biopsy operation, we acknowledge minimal errors could produce from the typical three stages of ROI delineation, navigation registration error, and brain shift of biopsy operation, which is unavoidable under our current study design.

## 6 Conclusion

Our study indicates that SEM-derived parameters DDC and α are significant in histological grading of gliomas, DDC may indicate proliferative ability, and CD34-stained microvascular perfusion may be an important determinant of water diffusion inhomogeneity α in gliomas. Therefore, our results assert the promise of SEM-diffusion imaging for future clinical practice of glioma.

## Data availability statement

The original contributions presented in the study are included in the article/supplementary material. Further inquiries can be directed to the corresponding authors.

## Ethics statement

The studies involving human participants were reviewed and approved by Huashan Hospital Ethics Committee. The patients/participants provided their written informed consent to participate in this study.

## Author contributions

HP and YR contributed to the conception and design of the study. JuW wrote the first draft of the manuscript. YR and HL critically revised the manuscript. HZ performed the data acquisition and analysis. XD and WR performed the clinical assessment. HC, JuW, YZ, TQ, ZY and HL and revised sections of the manuscript. All authors contributed to manuscript revision and approved the submitted version.
